# Dietary encapsulated fennel seed (*Foeniculum vulgare* Mill.) essential oil supplementation improves performance, modifies the intestinal microflora, morphology, and transcriptome profile of broiler chickens

**DOI:** 10.1093/jas/skae035

**Published:** 2024-02-08

**Authors:** Hasan Hüseyin İpçak, Ahmet Alçiçek, Muzaffer Denli

**Affiliations:** Department of Animal Science, Faculty of Agriculture, Dicle University, Diyarbakır 21280, Turkey; Department of Animal Science, Faculty of Agriculture, Ege University, İzmir 35100, Turkey; Department of Animal Science, Faculty of Agriculture, Ege University, İzmir 35100, Turkey; Department of Animal Science, Faculty of Agriculture, Dicle University, Diyarbakır 21280, Turkey

**Keywords:** broiler, essential oil, fennel seed, intestinal health, performance, transcriptome profile

## Abstract

Global antimicrobial resistance has led to a ban on the use of antibiotics as growth promoters (**AGPs**) in poultry farming, encouraging the use of natural phytogenic feed additives that provide similar effects to AGPs without causing resistance. The aim of this study was to determine the effects of the addition of encapsulated fennel seed (*Foeniculum vulgare* Mill.) essential oil (**FEO**) into the diets on the performance, intestinal microflora, morphology, and transcriptomic profiling of broiler chickens. In the study, 400 one-d-old male chicks of the Ross-308 genotype were randomly distributed into five groups, each with 16 replicates of five birds. The experiment included a control group fed on basal diets without the addition of FEO and treatment groups supplemented with 50 (FEO50), 100 (FEO100), 200 (FEO200), or 400 (FEO400) mg of encapsulated FEO/kg. Body weight and the European Production Efficiency Factor values were higher in the FEO100, FEO200, and FEO400 groups (*P* < 0.05). The feed conversion ratio significantly improved at all FEO levels (*P* < 0.05). FEO supplementation improved duodenum, jejunum, and ileum morphologies. It enhanced mucosal layer thickness in the duodenum and jejunum, and muscular layer thickness in the jejunum and ileum (*P* < 0.05). It also increased the number of *Lactobacillus* spp. in the jejunum and ileum (*P* < 0.05). According to the transcriptome profile obtained from the microarray analysis of samples taken from small intestine tissues, the mRNA expression levels of 261 genes in the FEO50 group (206 upregulated and 55 downregulated), 302 genes in the FEO100 group (218 upregulated and 84 downregulated), 292 genes in the FEO200 group (231 upregulated and 61 downregulated), and 348 genes in the FEO400 group (268 upregulated and 80 downregulated) changed compared to the control group. Most upregulated genes were associated with catalytic activity, binding, transcription regulators and transcription factors, anatomical structure and cellular development, and protein binding activity modulators. The downregulated genes mostly belonged to the transporter, carrier, and protein-modifying enzyme classes. Besides, the anti-inflammatory IL-10 gene (4.41-fold) increased significantly in the FEO100 group compared to the control group (*P* < 0.05). In conclusion, FEO improved the performance of broiler chickens by regulating biological processes such as performance and intestinal health, with the 100 mg FEO/kg supplementation being the most prominent.

## Teaser

The optimal level of 100 mg encapsulated fennel seed essential oil per kilogram of diet significantly boosted growth, gut health, and transcriptomic responses in broiler chickens.

## Introduction

The widespread use of antibiotics as growth promoters (**AGPs**) in poultry since the late 1940s has enhanced growth and feed efficiency but has led to the rise of antibiotic-resistant bacteria, posing a public health threat. This concern resulted in the ban on AGPs in 2006, increasing dependence on poultry immune systems and sparking a surge in therapeutic antibiotic use (Casewell et al., 2003; Brenes and Roura, 2010). Thus, there has been a significant focus on developing natural and effective alternatives to AGPs that do not promote antimicrobial resistance or maintain poultry health and performance. Phytogenic feed additives (**PFAs**) have emerged as the most extensively researched category in the field of poultry production over the past two decades. However, to develop an ideal alternative to AGPs, it is necessary to individually examine each compound in the phytogenic class and determine their specific activities in poultry. Fennel (*Foeniculum vulgare* Mill.) is an aromatic, edible plant with yellow flowers and feathery leaves, belonging to the Apiaceae (Umbelliferaceae) family (Abd el-Hack et al., 2020; Anka et al., 2020). Fennel has been used as an herbal medicine in traditional and alternative medicine for many years (Ghasemian et al., 2020). It possesses various biological and pharmacological activities, including antibacterial, antioxidant, galactogenic, estrogenic, anticancer, antitumor, apoptotic, hepatoprotective, antiviral, antiulcer, anti-inflammatory, and anticolitic effects (Rather et al., 2012; Badgujar et al., 2014; Diao et al., 2014; Kooti et al., 2015; Rani and Das, 2016). However, the effects of fennel can vary depending on the presence and variety of numerous valuable phytochemical compounds in its structure, including volatile components, flavonoids, hydrocarbons, phenolic components, fatty acids, and amino acids. Researchers have found the highest amounts of phytochemical components in fennel seed essential oil (FEO) (Badgujar et al., 2014).

The primary volatile component of FEO is trans-anethole, which is mainly responsible for its antimicrobial activity in organisms (Kubo et al., 2008; Shahat et al., 2011; Ghasemian et al., 2020). Yu et al. (2021) found that the addition of different levels of trans-ethanol to diets increased the average daily feed intake (**FI**) of broiler chickens but had no effect on average daily gain, feed/gain, and body weight over the entire experimental period, whereas 400 mg/kg trans-ethanol increased villus height (**VH**), crypt depth (**CD**), VH:CD, and *Bifidobacterium* populations and decreased the *Escherichia coli* population. Ghiasvand et al. (2021) reported that 200 mg/kg FEO did not affect the performance of broiler chickens during the entire period but decreased ileal *E. coli* and *Lactobacillus* spp. populations. There have been numerous in vitro studies on FEO’s antimicrobial, antimycobacterial, and antiviral potential. FEO also demonstrated antibacterial effects against food-borne pathogens such as *E. coli* 0157: H7, *Listeria monocytogenes* and *Staphylococcus aureus* (Cantore et al., 2004), *Bacillus megaterium*, *E. coli*, and *S. aureus* (Mohsenzadeh, 2007). In vivo studies investigating the effects of FEO on broiler chickens have primarily focused on humoral and cellular immune responses, and antioxidant defense against stress factors originating from temperature and *Eimeria* spp. (Mohammed and Abbas, 2009; Ragab et al., 2013; Safaei-Cherehh et al., 2018). Furthermore, Ghasemian et al. (2020) reported that FEO is rich in anisaldehyde, which interferes with cellular molecular targets. Yu et al. (2021) found that 400 mg/kg trans-anethole increased the mRNA expression of jejunal and ileal Na^+^/glucose co-transporter (SGLT1), oligopeptide transporter 1 (PepT1), ileal occludin (OCLN), claudin-1 (CLDN-1), and mucin 2 (MUC2). Another study conducted by the same research group reported that trans-anethole alleviated subclinical NE infection in broiler chickens with subclinical necrohemorrhagic enteritis (**NE**) by restoring intestinal barrier integrity, inhibiting the NF-kB signaling pathway, and modulating intestinal microbiota (Yu et al., 2022). Kim et al. (2013a) used microarray hybridization for gene expression profiling and identified 1.810 transcripts (677 upregulated and 1.133 downregulated) in the intestinal lymphocytes of *Eimeria acervulina*-infected broiler chickens supplemented with 15 mg/kg anethole compared to control groups. The authors reported that the biological functions of the genes in the transcriptome were related to the inflammatory response.

However, the antimicrobial activities of essential oils can decrease owing to the absorption of feed components. Therefore, most essential oils may be lost in the gastrointestinal system before reaching the small intestine, where their primary effects are observed, or during feed processing (Yang et al., 2015). Moreover, because of their lipophilic nature, it is challenging to deliver these components to the intestine (Upadhaya and Kim, 2017). Hence, they require an effective carrier (Dhama et al., 2015). With the development of nanotechnology, this problem can be solved by using microencapsulation techniques (Yang et al., 2015). Mirzaei et al. (2023) found that FEO nanoemulsion increased the number of *Lactobacillus* and decreased the number of *Coliform* and *E. coli* in the secum compared to the control and fennel essential oil-supplemented groups. In the current study, researchers have also encapsulated FEO to convert it from liquid to powder form, creating more homogeneous feed mixtures and allowing its release as desired in the targeted digestive tract. Although in vitro studies have investigated the antibacterial, antifungal, antiviral, and antimicrobial activities of FEO, very few in vivo studies have investigated its mechanisms of action as an immunomodulator, such as promoting growth, improving feed conversion efficiency, enhancing overall gut health, and strengthening immunity. Therefore, this study aimed to determine the effect of adding different levels of encapsulated FEO, a potent antimicrobial, on the performance, intestinal microflora, and morphology of broiler chickens. We also examined the feed-gene interactions mediated by FEO through small intestine transcriptome profiling using Affymetrix GeneChip Array technology.

## Materials and Methods

### Birds, Housing, and Diets

This study was approved by the Dicle University Experimental Animal Ethics Committee (DÜHADEK) with the decision dated July 05, 2019, numbered 73439, and protocol number 2018/24. This experiment was conducted at an indoor broiler test coop located in the facilities of the Department of Animal Science, Faculty of Agriculture at Dicle University. A total of 400 one-day-old male broiler chicks of the Ross-308 genotype were obtained from a 33-wk-old breeder flock and a commercial hatchery operating in Adana, Türkiye. The experiment included five groups (control and four treatment groups), each containing 80 chicks. Each group was divided into 16 subgroups, with five birds in each subgroup and the experiment lasted for 42 d. In each pen, 5- to 8-cm thick wood shavings were laid as litter. The birds in each replicate (0.5 m × 1 m = 0.5 m^2^) were group fed and feed was provided ad libitum, with the amount of feed they could consume daily being kept in front of them at all times. Chick feeders were used in the first week, and then hanging rectangular feeders (10 cm wide × 40 cm long × 10 cm high) were used in the following weeks. Water was provided using a nipple-drinker system, with two dripper heads in each pen. During the experiment, a light cycle of 23 h of light and 1 h of darkness was applied. Lighting was performed using light bulbs at night. The coop was heated using electric heaters and ventilated using a fan (40 cm diameter) and five windows (20 cm width × 40 cm length). The temperature inside the coop was set at 32 to 34 °C for the first 5 d, and then gradually decreased and remained constant at a minimum of 22 to 24 °C for the following days. Also, 55% to 60% relative humidity was provided to the broiler coop. The temperature and humidity inside the broiler coop were monitored daily using a digital temperature and humidity meter (VZN, Türkiye).

The volatile components of FEO (≥99% Birlikas, Izmir, Türkiye) obtained by aqueous distillation were determined by gas chromatography–mass spectrometry (Agilent 5977A GC/MS, USA) (Aprotosoaie et al., 2008; Acimovic et al., 2015; Ghasemian et al., 2020). The fatty acid methyl esters (**FAME**) content of FEO was determined at the Central Research Test and Analysis Laboratory Application and Research Unit at Ege University (EGE-MATAL) by modifying the methods reported by Synowiec et al. (2017) and Diao et al. (2014) (Table 1).

**Table 1.** Volatile component and fatty acid composition of FEO

Volatile component	Quantity (%)	Fatty acids	Quantity (%)
α-Pinene	0.39	Butyric acid (C4:0)	0.02
Phenol	0.40	Caproic acid (C6:0)	0.18
β-Pinene	0.03	Caprylic acid (C8:0)	0.01
β-Phellandrene	0.03	Capric acid (C10:0)	0.02
β-Myrcene	0.06	Undecanoic acid (C11:0)	0.07
α-Phellandrene	0.06	Tridecanic acid (C13:0)	0.01
α-Terpinene	0.03	Myristic acid (C14:0)	1.48
Limonene	9.52	Myristoleic acid (C14:1)	0.08
Sabinene	0.09	Pentadecanoic acid (C15:0)	0.02
Methyleugenol	0.12	Palmitic acid (C16:0)	6.29
β-Ocimene	0.18	Palmitoleic acid (C16:1)	0.03
ϒ-Terpinene	0.10	Heptadecanoic acid (C17:0)	0.11
Benzen	0.41	Cis-10-heptadecanoic acid (C17:1)	0.03
Dillapiole	0.37	Stearic acid (C18:0)	2.35
α-Thujone	0.52	Oleic acid (C18:1 cis)	33.44
4-Methoxyphenylacetone	0.40	Linoleic acid (C18:2 cis)	54.09
Bicyclo[2.2.1]heptan-2-ol	0.18	Arachidic acid (C20:0)	0.27
Linalool	0.05	Linolenic acid (C18:3 n3)	0.10
Dihydrocarvone	0.23	Cis-11-Eicosenoic acid (C20:1)	0.16
Cyclohexanone	0.16	Heneicosanoic acid (C21:0)	0.06
Anisole	3.28	Cis-11, 14-Eicodadienoic (C20:2)	0.01
Carvone	3.13	Behenic acid (C22:0)	0.73
Cuminaldehyde	0.10	Arachidonic acid (C20:4)	0.01
Trans-anethole	75.38	Trichosanoic acid (C23:0)	0.03
Neophytadiene	0.03	Lignoceric acid (C24:0)	0.28
P-Anisaldehyde	2.95	Nervonic acid (C24:1)	0.01

The minimum inhibitory concentrations (**MIC**), minimum bactericidal (**MBC**), and fungicidal (**MFC**) concentrations of FEO were determined at the Microbiological Analysis Laboratory at Ege University (EGE-MİKAL) according to the modified CLSI-M07-A9 method (Table 2). To ensure homogeneous mixing of FEO in basal diets, encapsulation processes (Into Biotechnology Industry and Trade Co. Ltd., Türkiye) were carried out by trapping Na-alginate gel beads through a revision of the methods described by Reineccius (1991) and Gouin (2004). Based on the volatile component, FAME, MIC, MBC, and MFC results, the lowest FEO level was determined as 50 mg/kg. The study was carried out according to a random plot experimental plan and the treatment groups were as follows: 0 (Control, FEO0), 50 (FEO50), 100 (FEO100), 200 (FEO200), and 400 (FEO400) mg of encapsulated FEO added to each kg of basal diet.

**Table 2.** Minimum inhibitory and microbicidal concentrations of FEO.

Microorganisms	Minimum inhibitory concentrations											Minimum microbicidal concentration (ppm)
	FEO concentrations (ppm)											
	5.000	2.500	1.250	625	312,5	156,3	78,1	39,1	19,5	Pos.^1^	Neg.^2^	
*Staphylococcus aureus* *ATCC 6538*	−	−	+	+	+	+	+	+	+	−	+	2.500
*Enterobacter aerogenes* ATCC 13048	−	−	−	+	+	+	+	+	+	−	+	1.250
*Esherichia coli* ATCC 10536	−	−	−	−	−	−	−	+	+	−	+	78,1
*Pseudomonas aeruginosa* ATCC 15442	−	−	+	+	+	+	+	+	+	−	+	2.500
*Salmonella typhimurium* ATCC 13311	−	−	−	−	−	−	+	+	+	−	+	312,5
*Candida albicans* ATCC 10231	−	−	−	−	−	−	−	−	+	−	+	78,1

^1^Positive control (Mueller Hinton Broth + organism + Gentamicin/Cycloheximide).

^2^Negative control (Mueller Hinton Broth + organism).

+: reproduction present, −: no reproduction.

During the experiment, birds were fed broiler chick starter diet from days 1 to 21 and broiler chicken finisher diet from days 22 to 42. The diets of the experimental groups were prepared in mash form at the feed production facility of the Department of Animal Science, Faculty of Agriculture at Dicle University following the Ross-308 guidelines and the nutrient requirements of male broiler chicks and broiler chickens reported by the National Research Council (1994). The proximate composition of the major ingredients in the diets was analyzed before the experiment. The composition (g/kg), nutrient content (%), and metabolizable energy (kcal ME/kg) levels of the diets used in this study are listed in Table 3. In addition, the nutrient contents of the diets used in the trial were determined using the Weende analysis method (AOAC, 2000). The sugar levels in the diets were determined using the Luff–Schoorl technique, as described in the TS 12232 standard, while starch content was measured using a two-step process outlined in the TS ISO 6493 standard (TSE, 1997, 2004). According to the communiqué (No.: 2004/33) published in the Official Gazette and reported by Hartel et al. (1977), ME, kcal/kg={[(0.1551 × CP, %) + (0.3431 × EE, %) + (0.1669 × Starch, %) + (0.1301 × Total Sugar, %)]/ 4.184} × 1.000 was calculated using the formula.

**Table 3.** Ingredients, composition (g/kg), nutrient content (%), and metabolizable energy (kcal/kg) levels of broiler starter and finisher diets.

Item	Starter diets (days 0 to 21)					Finisher diets (days 22 to 42)				
	FEO0	FEO50	FEO100	FEO200	FEO400	FEO0	FEO50	FEO100	FEO200	FEO400
Corn	556.00	556.00	556.00	556.00	556.00	570.00	570.00	570.00	570.00	570.00
Soybean meal (46% CP)	283.00	283.00	283.00	283.00	283.00	227.00	227.00	227.00	227.00	227.00
Full fat toasted soybeans (36 % CP)	120.00	120.00	120.00	120.00	120.00	120.00	120.00	120.00	120.00	120.00
Bone meal (27 % Ca, 18 % CP)	20.00	20.00	20.00	20.00	20.00	30.00	30.00	30.00	30.00	30.00
Limestone (CaCO_3_)	4.50	4.50	4.50	4.50	4.50	4.00	4.00	4.00	4.00	4.00
Dicalcium phosphate	7.00	7.00	7.00	7.00	7.00	—	—	—	—	—
Sunflower oil (8,800 kcal/kg)	—	—	—	—	—	40.00	40.00	40.00	40.00	40.00
dl-Methionine	1.50	1.50	1.50	1.50	1.50	1.50	1.50	1.50	1.50	1.50
l-Lysine	0.50	0.50	0.50	0.50	0.50	1.00	1.00	1.00	1.00	1.00
Vitamin—mineral premix^1^	3.50	3.50	3.50	3.50	3.50	3.00	3.00	3.00	3.00	3.00
NaCl	4.00	4.00	4.00	4.00	4.00	3.50	3.50	3.50	3.50	3.50
Encapsulated FEO	—	0.05	0.10	0.20	0.40	—	0.05	0.10	0.20	0.40
Analyzed chemical composition (%)										
Dry matter	89.68	89.59	89.61	89.64	89.43	90.05	89.72	89.76	90.10	89.90
Crude protein	22.52	22.50	22.59	22.51	22.53	20.64	20.58	20.62	20.63	20.60
Ether extract	7.81	7.75	7.65	7.76	7.69	10.10	10.20	10.14	10.22	10.20
Crude fiber	3.26	3.20	3.22	3.29	3.31	3.80	3.72	3.75	3.72	3.81
Crude ash	5.60	5.80	5.65	5.90	5.45	5.71	5.80	5.80	5.64	5.90
Starch	36.24	36.43	36.54	36.28	36.60	38.04	38.06	38.14	38.10	37.90
Sugar	4.76	4.64	4.75	4.76	4.72	4.20	3.98	4.10	3.95	4.18
Metabolizable energy (ME poultry), kcal/kg	3.068,88	3.067,07	3.070,01	3.066,01	3.072,53	3.241,36	3.241,30	3.244,78	3.245,45	3.241,88
Calculated values										
Lysine, %	1.31	1.31	1.31	1.31	1.31	1.19	1.19	1.19	1.19	1.19
Methionine + cystine	0.90	0.90	0.90	0.90	0.90	0.82	0.82	0.82	0.82	0.82
Ca, %	1.00	1.00	1.00	1.00	1.00	1.07	1.07	1.07	1.07	1.07
Available P	0.54	0.54	0.54	0.54	0.54	0.53	0.53	0.53	0.53	0.53

^1^Vitamin and mineral premix provided per 2.5 kg of diet: vitamin A, 12.000.000 IU; vitamin D3, 1.500.000 IU; vitamin E, 40.000 mg; vitamin K3, 5.000 mg; vitamin B1, 3.000 mg; vitamin B2, 7.000 mg; vitamin B6, 5.000 mg; vitamin B12, 30 mg; CAL-D pantothenate, 10.000 mg; Biotin, 75 mg; Folic acid, 1.000 mg; Niacin amide, 4.000 mg; Choline chloride, 400.000 mg; Mn sulfate, 80.000 mg; Fe (II) sulfate, 60.000 mg; Cu (II) sulfate, 5.000 mg; Zn sulfate, 60.000 mg; Ca iodide, 1.000 mg; Na selenite, 150 mg, and CaCO_3_ 1.135.000 mg.

The body weights (**BWs**) of individual birds and feed leftovers were recorded weekly on days 0, 21, and 42. Body weight gain (**BWG**), FI, and feed conversion ratio (**FCR**, adjusted for mortality and calculated as total FI divided by total gain including the weight of lost birds), and mortality were measured on days 21 and 42. At the end of the study, the European Production Efficiency Factor (**EPEF**) value of each subgroup was calculated using the formula: [viability days 0 to 42 (%) × BW at day 42 (kg) × 100]/[age (d) × FCR days 0 to 42].

### Sampling procedures

At the end of the experiment, 10 birds per group, closest to the mean BW of the group, were leg-banded, and their BWs were recorded before slaughter. The birds were exsanguinated via the left carotid artery before decapitation and allowed to bleed for approximately 2 min. After bleeding, the abdomen was opened, and the internal organs were removed. For small intestinal microbiota composition, jejunal and ileal contents were quickly removed and kept in at −20 °C. All small intestine samples were washed with isotonic water and divided into segments (duodenum, jejunum, and ileum) for histomorphological analysis. Subsequently, 1 cm tissue sections were taken from the duodenum 8 to 10 cm distal to the beginning of the small intestine, from the jejunum 8 to 10 cm proximal to Meckel’s diverticulum, and from the ileum 8 to 10 cm proximal to the ileocecal junction. Then, these samples were placed in falcon tubes containing 10% formalin (formaldehyde 37%, Isolab, Wertheim, Germany). For small intestine transcriptome profiling, 2 to 3 cm sections were taken from each segment of the small intestine of birds, and tissue pools were formed. These tissue pools were placed separately in 15 mL RNase/DNase-free falcon tubes, labeled, kept in dry ice at −80 °C transported to the laboratory, and stored at −80 °C until analysis. The weights of the carcass, thigh meat, breast meat, abdominal fat, liver, heart, spleen, pancreas, bursa fabricius, bezel stomach, and gizzard, as well as the total weight of small intestine and large intestine (before segmentation) of the birds, were recorded. Relative organ weights were calculated by proportioning to the preslaughter BW of the birds. Carcass yield was calculated by dividing the hot carcass weight by the preslaughter BW.

### Histomorphological Analysis

Small intestinal segments were fixed in 10% formalin for 24 hours, and after fixation, the tissues were washed under low running water for 1 night to remove the fixative. Moreover, to remove formaldehyde, the tissues were washed with phosphate-buffered saline. Tissues were then dehydrated by immersion in different concentrations of ethyl alcohol (Merck, Germany). The tissues were transparentized by controlled immersion in xylene (≥ 98% Tekkim, Türkiye), and subsequently, they were placed in paraffin baths at 58 to 60 °C for three times at 1-h intervals and kept overnight in the last paraffin dish. The next day, the tissues were embedded in paraffin in metal molds and blocks were prepared. After the frozen paraffin blocks were trimmed, and 4 to 5 μm thick sections were taken using a rotary microtome device (Leica, USA). These sections were then taken on slides and stained with hematoxylin–eosin (Gurr, 1972; Sakamoto et al., 2000).

For measurement purposes, the stained sections were examined under a light microscope (ZEISS Axio Imager A2, USA) and photographed with a camera (ZEISS Axicom 208 color) integrated into the light microscope. Tunica mucosa thickness (mucosal layer, **TMT**) and muscularis mucosa thickness (muscular layer, **MMT**) were measured using an image processing and analysis software (ZEN 3.0 lite photography software, Zeiss Microscopy, USA) as reported by Barrett et al. (2013). VH (from villus apex to crypt mouth), CD (from crypt basal to crypt mouth), and villus width (**VW**, from villus basal) were measured as described by Boumphrey (2009) and Murugesan et al. (2015). Twenty measurements for each parameter per treatment group were considered. Villus area (**VA**, mm^2^) was calculated using the villus height/crypt depth ratio (VH/CD) and the [(2π×VH × (VW ÷ 2)] geometric model reported by Sakamoto et al. (2000).

### Determinations of intestinal microflora composition

For the detection of *E. coli* O-157:H7, *Salmonella* spp., *Clostridium perfringens*, *S. aureus*, and *Lactobacillus* spp. microorganisms by quantitative real-time polymerase chain reaction (qRT-PCR), deoxyribonucleic acids (DNAs) were isolated from each sample using the QIAamp DNA Stool Mini Kit (Qiagen Inc., Hilden, Germany). After isolation, the concentration and purity (OD260/280) of the DNAs were determined using a Nanodrop 2000 spectrophotometer (Thermo Scientific, Wilmington, DE, USA). The forward (F) and reverse (R) primers used for the microorganisms are listed in Table 4.

**Table 4.** Forward (F) and reverse (R) primers of microorganisms.

Microorganisms	Accession number	Primer name	Primer sequence (5ʹ to 3ʹ)	Primer position	Product length (bp)	Reference
*Clostridium perfringens*	JQ071556.1	F	ATGATTGGGATTATGCAGCAA	644-664	212	Fu et al., 2006
		R	TCCATCCTTTGTTTTGATTCCA	855-834		
*E. coli* *O-157:H7*	CP038425.1	F	GTCACAGTAACAAACCGTAACA	1543710-1543689	95	Jothikumar and Griffiths, 2002
		R	TCGTTGACTACTTCTTATCTGGA	1543616-1543638		
*Salmonella* spp.	CP051218.1	F	CTCACCAGGAGATTACAACAT	2718115-2718135	95	Bozkurt, 2018
		R	AGCTCAGACCAAAAGTGACCA	2718209-2718189		
*Staphylococcus aureus*	CP050690.1	F	AATTAACGAAATGGGCAGAAACA	1663950-1663972	94	Chowdhury et al., 2019
		R	TGCGCAACACCCTGAACTT	1664043-1664025		
*Lactobacillus* spp.	MG827407.2	F	GAGGCAGCAGTAGGGAATCTTC	363-384	126	Murri et al., 2013
		R	GGCCAGTTACTACCTCTATCCTTCTTC	488-462		

qRT-PCR analysis was performed on a Roche LightCycler 480 II system (Roche Diagnostics, Basel, Switzerland). Briefly, 2.5 μL DNA template, 5 μL LightCycler 480 SYBR Green I Master Mix, 1.9 μL PCR grade water, 0.3 μL F primer, and 0.3 μL R primer (10 μmol/L each) were added to a total volume of 10 μL. The amplification conditions were as follows:1) initial denaturation at 95 °C for 5 min; 2) 45 cycles of denaturation at 95 °C for 10 s, annealing at 55 °C for 15 s, and elongation at 72 °C for 10 s; 3) dissociation (melting curve analysis from 60 °C to 97 °C at 2.5 °C increments every 2.5 s); and cooling at 40 °C for 30 s. The LightCycler 480 II software was used for data analysis. For the detection and quantification of microorganism presence/absence, positive samples were detected using Amplification Curves with Cycle Threshold (Ct) values and a standard curve (constructed with samples with known plasmid copy number). The accuracy of the results was verified through a melting curve analysis (Tm).

### Total RNA isolation and processing of samples for microarray analysis

Total RNAs were isolated from small intestine tissues that were disintegrated by a homogenizer using the TriPure Isolation Reagent Kit (Roche Diagnostics, Switzerland). Ten pools were produced, consisting of equivalent amounts of small intestine samples mixed according to the control and supplemented groups. Each experimental group resulted in ten samples combined in two pools, which were RNA extracted for hybridization in a microarray. Changes in gene expression were analyzed by microarray technology using an Affymetrix GeneChip Chicken Genome Array, which contains 37.703 probe sets of 25 base lengths from Gallus gallus. Briefly, the total RNA from each sample was processed with a GeneChip WT PLUS Reagent Kit (Thermo Fisher Scientific, United States), labeled, fragmented, hybridized, and scanned using a GeneChip Chicken Genome Array according to the manufacturer’s instructions.

After microarray analysis, the experimental quality and hybridization controls of gene expression data were evaluated with benchmark values and graphs using the Affymetrix Transcriptome Analysis Console (**TAC**) 4.0 software. For this purpose, normalization methods like the Robust Multiarray Analysis (**RMA**) algorithm and the Signal Space Transformation-Robust Multiarray Analysis (**SST-RMA**) method were used. The success of the normalization step is shown with box plots and all samples in the analysis were considered “successful”.

### Gene ontology analysis

Raw data files in the CEL format were obtained from the analysis using the TAC 4.0 software package. At the end of the microarray analysis, when the gene expression values were analyzed among the experimental groups, many genes expressed at different levels were obtained. After the log_2_ transformation of gene expression values of the FEO50, FEO100, FEO200, and FEO400 groups, gene lists showing fold changes were created. Then, multivariate statistical analysis was applied using principal component analysis (**PCA**) for the observed differences between the experimental groups. In addition, a comparative analysis with the reference genome of “Gallus gallus” was performed using Protein Analysis Through Evolutionary Relationships (PANTHER) version 15.0, to obtain comprehensive information about the functions of the genes. After the differentially expressed gene lists of the FEO-supplemented groups were uploaded to the PANTHER classification system, molecular function, biological process, and protein classifications were performed using Fisher’s exact statistical test method. Only the genes with a log fold change greater than or equal to 1.2-fold for upregulated genes and lower than or equal to −1.2-fold for downregulated genes were screened out as differentially expressed genes.

### Quantitative real-time PCR assays

To confirm the microarray analysis findings, regulated genes in the small intestine tissue, including interleukin 10 (IL-10), mucin 2 (MUC2), claudin 5 (CLDN5), and caspase 3 (CASP3), were selected for further validation by qRT-PCR. Total RNA samples were isolated from the small intestine of 10 birds from each treatment group and 50 birds in total and stored them at −80 °C. Total RNA was used for cDNA synthesis using a BIORAD iScript cDNA Synthesis Kit (Bio-Rad Lab., USA). The reaction mixture consisted of 10 μL H_2_O, 4 μL 5× iScript Reaction Mix, 1 μL iScript Reverse Transcriptase, and 5 μL ribonucleic acid (RNA) sample, with a total volume of 20 μL for a single sample. The Thermocycler (Bio-Rad Lab., USA) protocol was conducted as follows: priming (25 °C for 5 min), Reverse Transcription (46 °C for 20 min), RT Inactivation (95 °C for 1 min), and holding at 4 °C. The samples were then stored at −20 °C until further use. Primers for IL-10, MUC2, CLDN5, and CASP3 target genes and beta-actin housekeeping gene regions were designed using the NCBI and ENSEMBL gene banks (Table 5), and the specificity of the primers was checked using the BLAST program. Real-time PCR analysis was performed using a Roche LightCycler 480 II (Roche Diagnostics, Switzerland), and the reaction mixture (10 μL) contained 2.5 μL cDNA, 5 μL LightCycler 480 SYBR Green I Master Mix, 1.9 μL PCR grade water, 0.3 μL F primer and 0.3 μL R primer (10 μmol/L each). The experimental run protocol was as follows: initial incubation for 5 min at 95 °C; quantification program consisting of 45 cycles of denaturation at 95 °C for 10 s, annealing at 56 °C for 15 s, and elongation at 72 °C for 10 s; and ending with a melting curve analysis from 61 to 97 °C with 2.5 °C increment every 2.5 s, and cooling at 40 °C for 30 s. The Absolute Quantification and Advanced Relative Quantification methods were used for data analysis, and the fold change method was utilized in the results (Lee et al., 2006). With this method, target gene Ct values were normalized to housekeeping gene Ct values and fold-change values were determined by proportioning normalized values with the control group.

**Table 5.** Forward (F) and reverse (R) primers of genes

Genes^1^	Accession number	Primer name	Primer sequence (5ʹ to 3ʹ)^2^	Primer position	Product length (bp)
IL-10	NM_001004414.2	F	AGCTGAGGGTGAAGTTTGAG	116-135	97
		R	AACTCATCCAGCAGTTCAGAG	212-192	
MUC2	JX284122.1	F	CAGCACCAACTTCTCAGTTCC	3474-3494	102
		R	TCTGCAGCCACACATTCTTT	3575-3556	
CLDN5	NM_204201.1	F	GAGGGACCATCTACATCCTCT	458-478	86
		R	GTCGTAGAAGTCGCTGATGAC	543-523	
CASP3	NM_204725.1	F	ACAATGATCTGTCAAGCAGAGATA	457-480	99
		R	GGCTTAGCAACACACAAACAA	555-535	
ACTB	NM_205518.1	F	TGGGCCAGAAAGACAGCTAC	208-227	82
		R	CCGTGTTCAATGGGGTACTT	289-270	

^1^IL-10, interleukin 10; MUC2, mucin 2; CLDN5, claudin 5; CASP3, caspase 3; ACTB, beta-actin.

^2^All primers were designed.

### Statistical analysis

Statistical analyses of the obtained data were performed using SPSS (version 22.0; SPSS, 2013). The Shapiro–Wilk test was used to check for normality, and Levene’s test was applied to assess the homogeneity of variances. Differences between the mean values for groups were calculated via a one-way analysis of variance (**ANOVA**). Duncan’s test was used to check the significance of differences between groups, and the significance levels were determined as *P* < 0.05 or *P* < 0.01. Orthogonal contrasts were applied to explore linear and quadratic effects of the main factor as well. For the intestinal microflora data, the nonparametric Kruskal–Wallis test was performed based on a significance level of *P* < 0.05. Finally, the chi-squared test was conducted to check for differences between groups in terms of mortality rates.

## Results

### Broiler performance

The effects of different FEO supplementation levels on BW, BWG, FI, FCR, mortality, and EPEF are shown in Table 6. At the end of the starter period, BW values were similar across all FEO-supplemented groups but higher than the control groups (*P* < 0.01). At the end of the experiment, BW values increased in parallel with the increase in FEO levels and BW was highest in the FEO400 group and lowest in the control group (*P* < 0.01). The linear effect of increasing FEO levels on BW was significant on days 21 and 42 (*P* < 0.01). In addition, BW increased by 3.43%, 4.0%, 4.95%, and 6.37%, respectively, with an increase in the FEO level compared to the control group on day 42. Also, the linear effect of increasing FEO levels on BWG was significant during the starter period (*P* < 0.01). In the finisher phase, BWG values did not differ significantly between the experimental groups (*P* > 0.05). Overall, BWG increased with increasing FEO levels (linear effect, *P* < 0.01). However, FEO did not affect FI at any period (*P* > 0.05). FCR improved in the experimental groups during the starter period (1.56 *vs*. 1.37; *P* < 0.05), with a significant linear effect with increasing FEO levels (*P* < 0.01). In the finisher period, FCR values differed significantly between the experimental groups (linear effect, *P* < 0.05). In the total period, FCRs of broiler chickens fed all FEO-supplemented groups were significantly improved compared to the control group, and this effect was linearly related (1.74, 1.65, 1.64, 1.61, and 1.62 respectively; *P* < 0.05). In addition, FCR improved by 5.17%, 5.74%, 7.47%, and 6.90%, respectively, in parallel with the increase in FEO levels compared with the control group on day 42. Mortality rates did not show significant differences between the experimental groups over the total period (*P* > 0.05). EPEF values were significantly higher in the supplemented groups (*P* < 0.05), with a significant linear effect with increasing FEO levels (333.74 vs. 375.98; *P* < 0.05).

**Table 6.** Effect of FEO supplementation on BW, BWG, FI, FCR, mortality, and EPEF value in broiler chickens.

Item	FEO^1^ (mg/kg)					Pooled SEM^2^	*P* value		
	0	50	100	200	400		ANOVA	Linear	Quadratic
Starter period (days 0 to 21)									
BW day 0, g	39.38	39.32	39.25	39.13	39.30	0.166	0.993	0.774	0.771
BW day 21, g	594.80^b^	655.50^a^	648.60^a^	656.30^a^	678.46^a^	5.086	<0.001**	<0.001**	0.128
BWG, g/bird	555.43^b^	616.30^a^	604.23^ab^	609.28^ab^	639.16^a^	9.110	0.054	0.006**	0.544
FI, g/bird	848.73	883.00	869.26	858.78	867.95	6.266	0.514	0.186	0.375
FCR, g/g	1.56^b^	1.45^ab^	1.41^a^	1.40^a^	1.37^a^	0.021	0.026*	0.002**	0.197
Finisher period (days 22 to 42)									
BW day 42, g	2458.28^b^	2542.64^ab^	2556.51^a^	2579.93^a^	2614.85^a^	13.941	0.007**	0.002**	0.442
BWG, g/bird	1864.68	1887.36	1898.71	1931.83	1935.72	14.465	0.478	0.070	0.897
FI, g/bird	3329.36	3252.80	3258.29	3228.31	3262.48	20.927	0.627	0.136	0.300
FCR, g/g	1.80^b^	1.72^ab^	1.74^ab^	1.67^a^	1.71^ab^	0.016	0.177	0.034*	0.314
Total period (days 0 to 42)									
BWG, g/bird	2420.10^b^	2503.66^ab^	2507.66^ab^	2541.11^ab^	2574.88^a^	18.885	0.110	0.010**	0.651
FI, g/bird	4178.09	4135.80	4116.17	4087.29	4130.43	22.790	0.802	0.297	0.406
FCR, g/g	1.74^b^	1.65^a^	1.64^a^	1.61^a^	1.62^a^	0.014	0.026*	0.002**	0.111
Mortality, %	2.5	1.25	3.75	1.25	3.75	-	0.726	-	-
EPEF^3^	333.74^b^	363.57^ab^	371.09^a^	378.38^a^	375.98^a^	5.08	0.023*	0.028*	0.112

^1^The control group was fed basal diets without the addition of FEO0, and the treatment groups were fed basal diets supplemented with 50, 100, 200, and 400 mg encapsulated FEO/kg (FEO50, FEO100, FEO200, and FEO400, respectively).

^2^Pooled standard error of the mean.

^3^European Production Efficiency Factor, EPEF: [viability days 0 to 42 (%) × BW at day 42 (kg) × 100]/[age (d) × FCR days 0 to 42].

Mean values with different superscripts (^a,b^) within the same row are statistically different at *P < 0.05 and/or **P < 0.01.

### Carcass traits and organ weights

The effects of FEO on slaughter weight (**SW**), carcass traits, and organ weights and their ratios to SW percentage are given in Table 7. The mean SW values were similar across all experimental groups but higher than in the control group (*P* < 0.05). This showed a significant linear effect with increasing FEO levels (*P* < 0.01). The mean carcass weight was higher in FEO-supplemented groups, with a significant linear correlation (*P* < 0.01). The lowest carcass yield (70.14%) was observed in the control group while the highest carcass yield (72.05%) was seen in the FEO400 group (*P* < 0.05). Hence, increasing FEO levels had a significant linear effect on carcass yield (*P* < 0.01). Relative breast meat weight was similar across all FEO-supplemented groups but higher than the control group (*P* < 0.05), again with a significant linear correlation (*P* < 0.01). Relative abdominal fat weight was the lowest in the FEO50 group (0.96%), followed by the control group (1.09%) (linear effect, *P* < 0.05). Relative liver weight also increased significantly with higher FEO levels (linear effect, *P* < 0.05). Relative heart weight was highest in the FEO400 group (*P* < 0.05). Finally, increasing FEO levels had a significant linear effect on relative bursa of fabricius weight (*P* < 0.01).

**Table 7.** Effect of FEO supplementation on carcass traits and organ weights.

Item	FEO^1^ (mg/kg)					Pooled SEM^2^	*P* value		
	0	50	100	200	400		ANOVA	Linear	Quadratic
Slaughter weight, SW, g	2455.50^b^	2540.78^ab^	2592.30^a^	2608.00^a^	2642.20^a^	18.934	0.011*	0.002**	0.333
Carcass weight, g	1723.09^b^	1824.28^a^	1850.39^a^	1887.76^a^	1904.09^a^	14.850	<0.001**	<0.001**	0.120
Carcass yield, %	70.14^b^	71.15^ab^	71.28^ab^	71.41^ab^	72.05^a^	0.204	0.048*	0.008**	0.652
Breast meat, g/kg SW	23.49^b^	24.46^ab^	25.12^ab^	25.15^ab^	25.68^a^	0.241	0.035*	0.006**	0.428
Leg meat, g/kg SW	20.46	20.06	19.92	19.98	19.91	0.114	0.484	0.165	0.394
Abdominal fat, g/kg SW	1.09^ab^	0.96^a^	1.27^b^	1.26^b^	1.28^b^	0.043	0.073	0.027*	0.952
Liver, g/kg SW	2.00^b^	2.08^ab^	2.13^ab^	2.32^a^	2.17^ab^	0.037	0.126	0.026*	0.288
Heart, g/kg SW	0.59^b^	0.60^b^	0.62^ab^	0.55^b^	0.69^a^	0.014	0.034*	0.542	0.105
Spleen, g/kg SW	0.13	0.14	0.11	0.11	0.12	0.005	0.205	0.507	0.571
Proventriculus, g/kg SW	0.41	0.39	0.43	0.42	0.42	0.007	0.492	0.781	0.753
Gizzard, g/kg SW	2.31	2.25	2.31	2.47	2.34	0.043	0.582	0.747	0.917
Bursa fabricius, g/kg SW	0.16^b^	0.17^b^	0.19^ab^	0.23^a^	0.21^a^	0.007	0.002**	0.004**	0.406
Small intestine, g/kg SW	2.89	2.88	2.86	2.89	2.90	0.012	0.906	0.732	0.384
Duodenum, g/kg SW	0.56	0.56	0.56	0.57	0.58	0.002	0.922	0.969	0.502
Jejunum, g/kg SW	1.20	1.19	1.18	1.20	1.20	0.011	0.907	0.806	0.404
Ileum, g/kg SW	1.14	1.13	1.13	1.14	1.14	0.005	0.899	0.731	0.360
Cecum, g/kg SW	0.50	0.48	0.49	0.51	0.52	0.009	0.950	0.820	0.501
Large intestine, g/kg SW	0.21	0.22	0.23	0.24	0.25	0.011	0.947	0.555	0.983

^1^The control group was fed basal diets without the addition of FEO0, and the treatment groups were fed basal diets supplemented with 50, 100, 200, and 400 mg encapsulated FEO/kg (FEO50, FEO100, FEO200, and FEO400, respectively).

^2^Pooled standard error of the mean.

Mean values with different superscripts (^a,b,c^) within the same row are statistically different at *P < 0.05 and/or **P < 0.01.

### Histomorphology of small intestine

The effects of FEO on the proximal small intestine morphology are presented in Table 8. VH was highest in the FEO400 group (*P* < 0.01), with a significant linear effect with increasing FEO levels (*P* < 0.01). The mean duodenal CD values decreased significantly with increasing FEO levels (linear effect, *P* < 0.05). The duodenal VH/CD ratio also increased with an increase in the FEO level, and the highest VH/CD ratio was observed in the FEO400 group (6.37 vs. 9.22 μm; *P* < 0.01). Similarly, duodenal VW significantly increased with higher FEO levels (*P* < 0.05). The duodenal VA was the highest in the FEO400 group, followed by the FEO200, FEO100, FEO50, and control groups (2.02, 1.75, 1.67, 1.62, and 1.54 mm^2^ respectively; *P* < 0.05). TMT was significantly higher in all FEO-supplemented groups than the control group (*P* < 0.01), with a significant linear (*P* < 0.05) and quadratic (*P* < 0.01) effects. Jejunum VH increased significantly in FEO-supplemented groups (*P* < 0.01), with significant linear (*P* < 0.01) and quadratic (*P* < 0.05) effects. Jejunum VW and VA significantly increased in all FEO-supplemented groups, again with significant linear and quadratic correlations (*P* < 0.01). Jejunum MMT was highest in the FEO200 group and lowest in the control group (*P* < 0.01). This value also showed significant linear and quadratic correlations with increasing FEO levels (*P* < 0.01). Similarly, the highest ileal VH was found in the FEO400 group (*P* < 0.01). It was also observed that ileal VA significantly increased in all FEO-supplemented groups compared with that in the control group, and this increase was linearly and quadratically related (*P* < 0.01). Finally, the FEO400 group showed a significantly higher ileal MMT value than the control group (28.35 and 36.38 μm respectively; *P* < 0.05).

**Table 8.** Effect of FEO supplementation on histomorphology of small intestine.

Item	FEO^1^ (mg/kg)					Pooled SEM^2^	*P* value		
	0	50	100	200	400		ANOVA	Linear	Quadratic
Duodenum									
VH, μm	1887.71^b^	1897.44^b^	1971.38^b^	1963.99^b^	2091.20^a^	19.27	0.001**	0.002**	0.292
CD, μm	306.05^b^	291.85^ab^	281.85^ab^	269.20^ab^	235.59^a^	9.854	0.180	0.018*	0.608
VH/CD, μm	6.37^b^	7.08^b^	7.45^b^	7.92^ab^	9.22^a^	0.256	0.003**	0.010**	0.505
VW, μm	258.17^b^	264.92^ab^	270.73^ab^	280.68^ab^	306.65^a^	6.684	0.141	0.016*	0.436
VA, mm^2^	1.54^b^	1.62^b^	1.67^ab^	1.75^ab^	2.02^a^	0.056	0.044*	0.034*	0.359
TMT, μm	426.48^b^	487.84^a^	537.93^a^	542.54^a^	505.67^a^	10.381	0.002**	0.001**	0.003**
MMT, μm	34.53	37.35	37.78	37.83	36.88	0.687	0.562	0.065	0.175
Jejunum									
VH, μm	1228.50^c^	1438.73^b^	1659.47^a^	1603.08^a^	1731.10^a^	33.360	<0.001**	<0.001**	0.034*
CD, μm	279.57	243.77	268.93	272.03	263.81	9.855	0.837	0.684	0.696
VH/CD, μm	5.95	5.94	6.11	6.05	6.63	0.182	0.757	0.794	0.556
VW, μm	201.22^b^	284.04^a^	286.35^a^	310.23^a^	283.68^a^	7.944	<0.001**	0.001**	0.001**
VA, mm^2^	0.79^b^	1.29^a^	1.48^a^	1.58^a^	1.56^a^	0.061	<0.001**	<0.001**	0.008**
TMT, μm	398.53	388.43	395.01	495.93	422.88	21.552	0.474	0.721	0.865
MMT, μm	21.92^d^	31.97^c^	33.04^cb^	38.59^a^	37.46^ab^	1.091	<0.001**	<0.001**	0.007**
Ileum									
VH, μm	1049.73^c^	1057.10^c^	1065.79^bc^	1103.96^b^	1175.09^a^	8.433	<0.001**	0.001**	0.004**
CD, μm	287.41	246.10	236.51	235.75	233.75	8.472	0.233	0.056	0.218
VH/CD, μm	4.35	4.53	4.92	5.25	5.22	0.177	0.434	0.217	0.752
VW, μm	193.65	197.79	194.64	230.70	222.17	6.509	0.223	0.362	0.802
VA, mm^2^	0.28^d^	0.66^c^	0.67^bc^	0.80^ab^	0.83^a^	0.033	<0.001**	<0.001**	0.001**
TMT, μm	332.93	341.37	376.95	387.22	396.18	15.726	0.641	0.289	0.853
MMT, μm	28.35^b^	29.84^b^	30.22^b^	32.09^ab^	36.38^a^	0.906	0.049*	0.121	0.332

^1^The control group was fed basal diets without the addition of fennel seed essential oil (FEO0), and the treatment groups were fed basal diets supplemented with 50, 100, 200, and 400 mg encapsulated FEO/kg (FEO50, FEO100, FEO200, and FEO400, respectively).

^2^Pooled standard error of the mean.

VH, villus height; CD, crypt depth; VH/CD, villus height/crypt depth ratio; VW, villus width; VA, villus area; TMT, thickness tunica mucosa (mucosal layer); MMT, thickness muscularis mucosa (muscular layer). Mean values with different superscripts (^a,b,c,d)^ within the same row are statistically different at *P < 0.05 and/or **P < 0.01.

### Microflora composition of small intestine

The effects of FEO on jejunal and ileal microflora compositions are shown in Table 9. *Escherichia coli* O-157:H7 count and *Salmonella* spp. count in the jejunum did not differ significantly between the experimental groups (*P* > 0.05). *Escherichia coli* O-157:H7 was not detected in the FEO100, FEO200, and FEO400 groups. *Clostridium perfringens* count in the jejunum was the highest in the control group, similar among the FEO50, FEO100, and FEO200 groups, but not detected in the FEO400 group (0.68 for control, and 0.34 log_10_ copies/g for FEO50, FEO100, and FEO200; *P* > 0.05). Similarly, the number of *S. aureus* in the jejunum was not detected in the FEO200 and FEO400 groups, and the difference between the other experimental groups was not significant (*P* > 0.05). The *Lactobacillus* spp. count was highest in the FEO400 group, followed by the FEO100, FEO200, FEO50, and control groups (6.21, 5.84, 5.59, 5.40, and 4.98 log_10_ copies/g, respectively; *P* < 0.05). Regarding *E. coli* O-157:H7, *Salmonella* spp., and *C. perfringens* in the ileum, there was no significant difference between the experimental groups (*P* > 0.05). *Staphylococcus aureus* in the ileum was partially detected in the control and FEO50 groups but not in the FEO100, FEO200, and FEO400 groups (*P* < 0.05). Finally, *Lactobacillus* spp. count in the ileum was highest in the FEO400 group, similar between the FEO50, FEO100 and FEO200 groups and lowest in the control group (6.72, 5. 78, 6.17, 6.20, and 5.26 log_10_ copies/g respectively; *P* < 0.05).

**Table 9.** Effect of FEO supplementation on microflora composition of small intestine.

Item	FEO^1^ (mg/kg)					Pooled SEM^2^	*P* value
	0	50	100	200	400		
Jejunum							
*E. coli O-157:H7	0.16	0.16	ND^3^	ND	ND	0.045	0.570
*Salmonella spp.	0.73	0.71	0.34	0.85	0.34	0.173	0.805
*Clostridium perfringens	0.68	0.34	0.34	0.34	ND	0.139	0.663
*Staphylococcus aureus	0.16	0.16	0.31	ND	ND	0.024	0.663
* Lactobacillus* spp.	4.98^b^	5.40^ab^	5.84^ab^	5.59^ab^	6.21^a^	0.138	0.047
Ileum							
*E. coli O-157:H7	ND	0.16	ND	ND	ND	0.031	0.406
*Salmonella spp.	1.08	0.38	0.76	ND	0.85	0.179	0.368
*Clostridium perfringens	1.03	1.03	1.03	0.34	0.43	0.178	0.474
*Staphylococcus aureus	0.62^b^	0.16^ab^	ND	ND	ND	0.066	0.008
* Lactobacillus* spp.	5.26^b^	5.78^ab^	6.17^ab^	6.20^ab^	6.72^a^	0.159	0.046

^1^The control group was fed basal diets without the addition of fennel seed essential oil (FEO0), and the treatment groups were fed basal diets supplemented with 50, 100, 200, and 400 mg encapsulated FEO/kg (FEO50, FEO100, FEO200, and FEO400, respectively).

^2^Pooled standard error of the mean.

^3^Not detected.

Mean values with different superscripts (^a,b^) within the same row are statistically different at *P < 0.05 and/or **P < 0.01.

### Transcriptome profile and mRNA expression of IL10, MUC2, CLDN5, and CASP3 genes in small intestine

**Figure 1.**
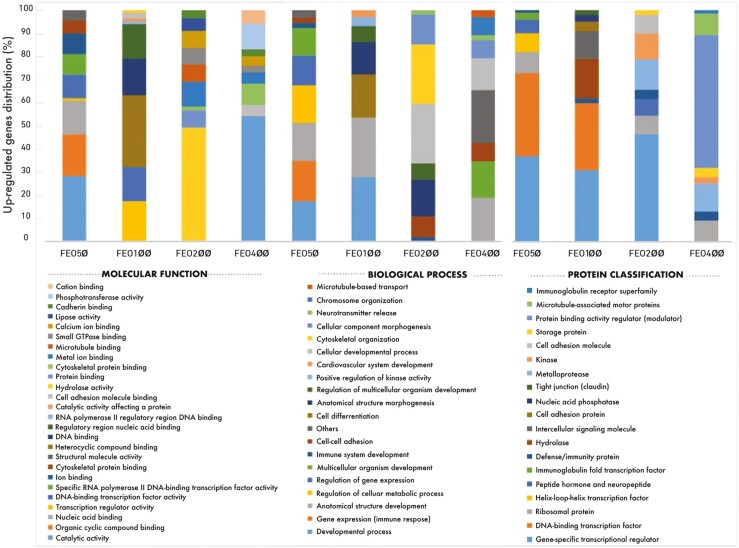
The distributions of molecular function, biological process, and protein classification of upregulated genes in the FEO supplemented groups (FEO50, FEO100, FEO200, and FEO400) compared to the control group (%).

Microarray analysis revealed that compared to the control group, the mRNA expression levels changed in 261 genes (206 upregulated, 55 downregulated) in the FEO50 group, 302 genes (218 upregulated, 84 downregulated) in the FEO100 group, 292 genes (231 upregulated, 61 downregulated) in the FEO200 group, and 348 genes (268 upregulated, 80 downregulated) in the FEO400 group. PCA revealed that the gene expression profiles of the FEO-supplemented groups differed significantly from the control group. The upregulated gene lists of the FEO-supplemented groups were uploaded to the PANTHER classification system, and the results of the molecular function, biological process, and protein classification of the genes are shown in Figure 1. Regarding the molecular function analysis of upregulated genes showed that most genes in the FEO50 group were classified as catalytic activity (25%), organic cyclic compound binding (16%), nucleic acid binding (13%), and transcription regulator activity (10%), etc.; most genes in the FEO100 group were involved in heterocyclic compound binding (25%), transcription regulator activity (14%), DNA binding (13%), DNA-binding transcription factor (**TF**) activity (12%), regulatory region nucleic acid binding (12%), RNA polymerase II regulatory region DNA binding (10%), and catalytic activity affecting a protein (10%), etc.; most genes in the FEO200 group were classified as protein binding (40%), hydrolase activity (27%), and cytoskeletal protein binding (10%), etc.; the majority of the genes in the FEO400 group were classified as catalytic activity (54%), and phosphotransferase activity (11%), etc.

Regarding the biological process of upregulated genes revealed that most genes in the FEO50 group were classified as a developmental process (16%), gene expression (immune response) (16%), anatomical structure development (15%), regulation of cellular metabolic process (15%), regulation of gene expression (12%), and multicellular organism development (11%), etc.; a vast majority of genes in the FEO100 group were characterized as a developmental process (28%), anatomical structure development (26%), cell differentiation (19%), and anatomical structure morphogenesis (14%), etc.; most genes in the FEO200 group were classified as a cellular developmental process (26%), cytoskeletal organization (26%), anatomical structure morphogenesis (16%), and cellular component morphogenesis (13%), etc.; most genes in the FEO400 group were classified as anatomical structure development (19%), multicellular organism development (16%), and cellular developmental process (14%), etc. With regards to the protein classification analysis of upregulated genes showed that most genes in the FEO50 group were classified as the gene-specific transcriptional regulator (37%), and DNA-binding transcription factor (36%); most genes in the FEO100 group were classified as the gene-specific transcriptional regulator (31%), DNA-binding transcription factor (29%), hydrolase (17%), and intercellular signaling molecule (12%), etc.; most the genes in the FEO200 group were classified as a gene-specific transcriptional regulator (46%), metalloprotease (13%), and kinase (11%), etc.; and most genes in the FEO400 group were classified as protein binding activity regulator (modulator) (43%), and kinase (20%).

**Figure 2.**
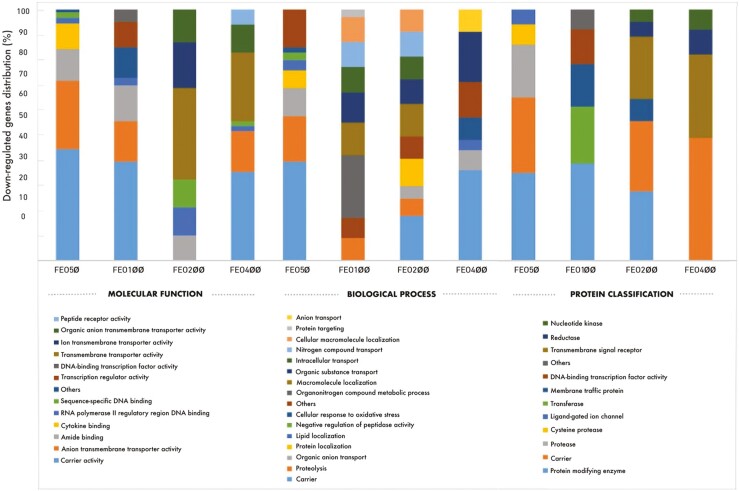
The distributions of molecular function, biological process, and protein classification of downregulated genes in the FEO supplemented groups (FEO50, FEO100, FEO200, and FEO400) compared to the control group (%).

The results of the molecular function, biological process, and protein classification of the downregulated gene lists in the FEO-supplemented groups are presented in Figure 2. Accordingly, the molecular function analysis of the downregulated genes showed that most genes in the FEO50 group were classified as carrier activity (39%), anion transmembrane transporter activity (24%), and amide binding (11%), etc.; most genes in the FEO100 group were classified as carrier activity (39%), anion transmembrane transporter (16%), amide binding (14%), and transcription regulator activity (10%), etc.; most genes in the FEO200 group were classified as transmembrane transporter activity (36%), ion transmembrane transporter activity (18%), organic anion transmembrane transporter activity (13%), RNA polymerase II regulatory region DNA binding (11%), sequence-specific DNA binding (11%), and amide binding (10%), etc; most genes in the FEO400 group were classified as carrier activity (35%), transmembrane transporter activity (27%), anion transmembrane transporter activity (16%), and organic anion transmembrane transporter activity (11%), etc.

Regarding the biological process of the downregulated genes showed that most genes in the FEO50 group were classified as the carrier (39%), proteolysis (18%), and organic anion transport (11%), etc.; most genes in the FEO100 group were expressed as the organonitrogen compound metabolic process (25%), macromolecule localization (13%), organic substance transport (12%), intracellular transport (10%), nitrogen compound transport (10%), and cellular macromolecule localization (10%), etc.; most genes in the FEO200 group were classified as the carrier (18%), macromolecule localization (13%), protein localization (11%), nitrogen compound transport (10%), organic substance transport (10%), etc.; most genes in the FEO400 group were classified as the carrier (36%), and organic substance transport (20%), etc. Finally, protein classification analysis of downregulated genes revealed that most genes in the FEO50 group were classified as protein-modifying enzymes (35%), carriers (30%), and proteases (21%), etc.; most genes in the FEO100 group were classified as protein-modifying enzymes (39%), transferases (23%), membrane traffic proteins (17%), and DNA-binding transcription factor activity (14%), etc.; most genes in the FEO200 group were classified as protein-modifying enzymes (28%), carriers (28%), and transmembrane signal receptors (25%), etc.; and most genes in the FEO400 group were in the classes of carriers (49%), transmembrane signal receptors (33%), and reductases (10%), etc.

**Figure 3.**
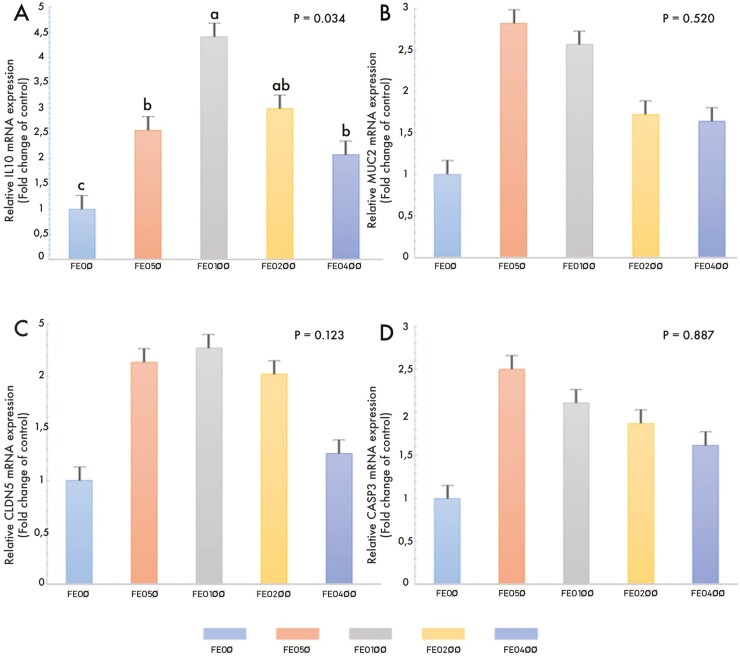
Effect of FEO supplementation on small intestinal mRNA expression levels: (A) IL-10, interleukin 10; (B) MUC2, mucin 2; (C) CLDN5, claudin 5; (D) CASP3, caspase 3. The control group was fed basal diets without the addition of FEO (FEO0), and the treatment groups were fed basal diets supplemented with 50, 100, 200, and 400 mg encapsulated FEO/kg (FEO50, FEO100, FEO200, and FEO400, respectively). Mean values with different superscripts (a,b,c) are statistically different at *P < 0.05.

The effects of FEO on the mRNA expression levels of IL10, MUC2, CLDN5, and CASP3 are given in Figure 3. Compared to the control group, the mRNA expression levels of the IL-10, which is an anti-inflammatory cytokine gene, were 4.41-fold higher in the FEO100 group, 2.99-fold higher in the FEO200 group, 2.56-fold higher in the FEO50 group, and 2.08-fold higher in the FEO400 group (*P* < 0.05). The mRNA expression levels of the MUC2, which is an oligomeric mucus gel former, increased 2.82-fold in the FEO50 group, 2.57-fold in the FEO100 group, 1.72-fold in the FEO200 group, and 1.64-fold in the FEO400 group (*P* > 0.05). The mRNA expression level of the CLDN5, which encodes a claudin protein that maintains intestinal integrity by establishing a strong connection between epithelial cells, increased 2.13-, 2.27-, 2.02-, and 1.26-fold in the FEO50, FEO100, FEO200, and FEO400 groups, respectively (*P* > 0.05). Finally, the mRNA expression levels of the CASP3, which plays an active role in regulating the immune system by activating apoptosis, increased 2.50-fold in the FEO50 group, 2.11-fold in the FEO100 group, 1.87-fold in the FEO200 group, and 1.62-fold in the FEO400 group (*P* > 0.05).

## Discussion

### Performance

According to our results, the addition of FEO to broiler diets significantly increased BW on days 21 and 42. Similarly, Mohammed and Abbas (2009) reported that fennel seeds increased broiler BW on day 42. Many PFAs have been used as alternatives to AGPs in broiler diets. Based on the relevant literature, coriander essential oil (Ghazanfari et al., 2015) and capsicum oleoresin, cinnamaldehyde, and carvacrol mixture (İpçak and Alçiçek, 2019) in broiler diets increased BW values. In contrast anise, ginger, and fennel species (El-Deek et al., 2002), essential oil mixtures (Çabuk et al., 2006), and oregano essential oil (Malayoğlu et al., 2010; Alp et al., 2012) did not affect BWs. Also, the addition of copaiba essential oil (Aguilar et al., 2013) and grape seed extract (Chamorro et al., 2013) reduced BW values. In the current study, FEO significantly and linearly increased BWG during the starter period and across the overall experiment. This result was consistent with the results of Cengiz et al. (2016) who reported higher BWG values with 100 mg/kg FEO supplementation. On the other hand, Safaei-Cherehh et al. (2018) found that fennel extract decreased BWG in all periods. In other studies, essential oil mixtures (oregano + laurel leaf + sage leaf + myrtle leaf + fennel seed + citrus peel oil) (Alçiçek et al. 2004), another plant extract mixture (thyme + peppermint + green tea and licorice extracts) (Attia et al., 2017), and carvacrol + thymol + limonene volatile component mixture (Hafeez et al., 2016) increased BWG, oregano essential oil (Alp et al., 2012) and fennel seed powder (Al-Sagan et al., 2020) did not change BWG values. Some researchers have found that oregano and garlic essential oil (Kırkpınar et al., 2011) reduced BWG values. Our positive findings regarding BW and BWG can be attributed to the antimicrobial activity of FEO, which reduces nutrient competition by destroying potentially pathogenic microorganisms, thus increasing nutrient and energy utilization for growth and development. Verstegen and Williams (2002) based a similar theory on a study by Vervaeke et al. (1979), suggesting that 6% of the net energy in pig diets may be lost due to bacterial utilization of glucose in the small intestine. Moreover, FEO as an immunoregulator can relieve broiler chickens from immune defense stress, such as pathogens or pathogen toxic metabolites (ammonia, biogenic amine, etc.), thereby increasing the intestinal availability that is necessary for nutrient absorption and helping birds grow more. Besides, FEO may have stimulated the release of endogenous enzymes, triggering BW gain in birds.

We found that FEO did not affect FI values at any phase of the experiment. Safaei-Cherehh et al. (2018) reported similar results regarding FI on days 0 to 21 and 22 to 42, but unlike our findings, they found reduced FI values at all FEO levels (100, 200, 300, or 400 ppm) in the overall period (days 0 to 42). Our FI results are consistent with those of Mohammed and Abbas (2009) and Cengiz et al. (2016), who found no change in FI values at any period. In other broiler research that used fennel spice (El-Deek et al., 2002) and fennel seed powder (Al-Sagan et al., 2020), FI values did not change either. However, in our study, FEO may not have affected feed palatability, and thus did not change FI, probably because it was encapsulated and showed its activity directly in the small intestine. Furthermore, we found that FEO significantly improved FCR at all periods, but there was no difference between the FEO levels. On day 42, FCR improved in parallel with increasing FEO levels. Similarly, Mohammed and Abbas (2009) found that fennel seed improved FCR on day 42. However, Cengiz et al. (2016) observed no changes in FCR with FEO, and Safaei-Cherehh et al. (2018) observed no changes with fennel extract. According to other studies, oregano essential oil (Alp et al., 2012), rosewood oil (Aguilar et al., 2014), ginger essential oil (Saleh et al., 2014), and plant extract mixture (Attia et al., 2017) improved FCR values; garlic essential oil (Kırkpınar et al., 2011) and fennel seed powder (Al-Sagan et al., 2020) did not change FCR values; oregano and thyme essential oil (Kırkpınar et al., 2011; Saleh et al., 2014) reduced FCR values. The positive effect of FEO on FCR is probably because it transforms the intestinal microbiota in favor of beneficial bacteria and changes the intestinal morphology to create more absorption on the surface area. In general, varying results with PFAs in broiler diets can be attributed to differences in type, origin, accession, harvest time, extraction method, composition of phytochemical components of the additive used and how it is fed to birds, level of inclusion, animal species, and environmental conditions.

EPEF is a performance criterion calculated by viability, BW, age, and FCR. Based on our findings, FEO supplementation increased EPEF values at ≥100 mg/kg levels and had no significant effect on mortality. This rise may be associated with increased BW and improved FCR values. In contrast to the study results, Ghiasvand et al. (2021) found that 200 mg/kg FEO significantly decreased the EPEF value, but Mirzaei et al. (2023) found that FEO nanoemulsion did not affect the EPEF value. Abd El-Ghany and Ismail (2014) reported that the addition of oregano oil to drinking water decreased EPEF values compared to the negative control group but increased it compared to the positive control group. Similar to our study, Petričević et al. (2018) found that adding 0.2% rosemary powder did not change EPEF values, but the 0.4% and 0.6% levels yielded higher values. Regarding the effect of PFAs on mortality, Alçiçek et al. (2004) and Küçükyılmaz et al. (2012) reported that essential oil mixtures including fennel did not affect mortality. İpçak and Alçiçek (2019) reported similar results for capsicum oleoresin, cinnamaldehyde, carvacrol, and their mixture. Ragab et al. (2013) found that 1% and 2% fennel seeds reduced mortality. In conclusion, fast-growing broiler chickens raised under commercial conditions are in contact with each other in limited areas and are susceptible to common infectious and metabolic diseases, leading to increased mortality rates. Therefore, FEO prevented the increase in mortality rate by suppressing pathogenic microorganisms through its antimicrobial activity, strengthening immunity, and increasing EPEF values by FCR and BW. In general, these inconsistencies in performance measures observed in the studies may be attributed to differences in the main volatile components and ratios of essential oils included in the diets.

### Carcass traits and organ weights

In the present research, adding different levels of FEO to broiler diets increased slaughter weight, carcass weight, carcass yield, relative breast meat weight, and abdominal fat weight. However, FEO supplementation did not change leg meat weight. Regarding the effect of PFAs on carcass parameters in broiler chickens, El-Deek et al. (2002) reported that the combination of fennel with ginger or anise species did not affect carcass or abdominal fat weights. Al-Sagan et al. (2020) found that fennel seed powder did not change carcass or abdominal fat weights but increased relative breast meat weight. Khattack et al. (2014) reported that essential oil mixture did not change slaughter weight or carcass weight but increased breast weight. Attia et al. (2017) stated that plant extract mixture did not change carcass weight or abdominal fat weight. Criste et al. (2017) found that rosehip powder decreased carcass weight. Meimandipour et al. (2017) reported no effect on carcass weight with encapsulated nettle root, dill, and aloe vera extract. In the present study, FEO increased the relative liver, heart, and bursa fabricius weights. El-Deek et al. (2002) reported no effect on the heart, liver, spleen, or pancreas with different combinations of fennel with ginger or anise species. Mohammed and Abbas (2009) found that heart, spleen, bursa of fabricius, gizzard, and liver weights were not affected by fennel seed. Al-Sagan et al. (2020) reported similar results for gizzard and liver weights. Soltan et al. (2008) reported that anise seeds had no effect on heart, gizzard, or liver. Çakmak et al. (2017) concluded that sumac powder had no effect on liver, spleen, or gizzard weights. Accordingly, our results regarding the liver, heart, and bursa of fabricius weights are partially compatible with previous research. It was concluded that the efficacy of PFAs may vary depending on factors such as form, level, diet, and environmental conditions. In fact, in this study, it was thought that the administration of FEO in the encapsulated form increased its efficacy, owing to more homogeneous mixing with the feed, suppression of volatile properties, and greater access to the target area. Furthermore, it may have promoted overall growth and therefore the development of specific organs based on the biological activities of FEO that promote growth and development.

### Histomorphology of small intestine

In the present study, FEO supplementation in diets significantly increased duodenal VH, CD, the VH/CD ratio, VW, and VA. Based on the literature review, many PFAs caused different results in the intestinal histomorphology of broiler chickens. Garlic meal added to broiler diets increased VH and CD but reduced VH/CD ratio (Adibmoradi et al., 2006). Dried fermented ginger did not change VH or VA (Incharoen et al., 2010). Peppermint essential oil (200 or 400 mg/kg) did not change VH but increased CD and decreased VH/CD ratio at 400 ppm (Emami et al, 2012). PFA mixture and *Boswellia serrata* resin did not change VH, CD, or VH/CD ratios (Tsirtsikos et al., 2012; Kiczorowska et al., 2016). *Euphorbia hirta* (7.5 g/kg) increased VH, VH/CD, and VA but decreased CD (Hashemi et al., 2014). However, compared to these findings, a PFA that can be used as an alternative to antibiotics is expected to increase VH, VH/CD ratio, and VA but to reduce CD. Paiva et al. (2014) found that shorter intestinal villi resulted in poorer absorption and digestion, which they attributed to a smaller absorption area and the presence of fewer mature erythrocytes. A deeper CD indicates more rapid cell loss. Increasing VH and reducing CD results in a significantly increased VH/CD ratio, indicating the presence of mature erythrocytes and the efficiency of nutrient absorption (Paiva et al., 2014; Zeng et al., 2015b). On the other hand, nutrients are absorbed through the villi. An increase in VA leads to the enlargement of the absorption surface and increases the probability that more nutrients are retained by the chemosensors. Hence, it can be concluded that supplementation with 400 mg/kg FEO positively affected intestinal morphology. The increase in VH induced by FEO may be attributed to its ability to increase the expression of genes involved in anatomical development, as determined by microarray analysis. Windisch et al. (2008) reported that the phenolic groups in essential oils may act as hydrogen donors, which could lead to increased VH due to their antioxidant activities. Furthermore, FEO increased duodenal TMT but not MMT. Similarly, Hashemi et al. (2014) reported that *Euphorbia hirta* did not affect MMT values. Murugesan et al. (2015) found that a feed additive containing over 30 essential oils and phytogenic components reduced mucosal thickness. It has been suggested that the mucosal layer reduces the adhesion of pathogenic microorganisms and therefore stabilizes microbial eubiosis in the intestine (Jamroz et al., 2006). However, mucosal layers surround the intestinal epithelial cells, both increasing their surface area and protecting them against irritation by potentially pathogenic metabolites or chemicals. Thus, it can be said that the addition of FEO in broiler diets has a positive effect by stimulating intestinal mucus secretion.

In this study, FEO supplementation in broiler diets significantly increased jejunum VH, VW, VA, and MMT but did not change TMT. Similarly, Hashemipour et al. (2013) found that thymol + carvacrol (1:1) supplementation in broiler diets increased VH, VH/CD ratio, VA, and muscular thickness but did not change VW, CD, or mucosal thickness. Based on other research investigating the efficacy of PFAs on jejunum morphology, garlic meal increased VH, CD, and VH/CD ratio (Adibmoradi et al., 2006); essential oil mixture reduced VH and CD, whereas hydroalcoholic extract mixture did not (García et al., 2007); dried fermented ginger did not change VH or VA (Incharoen et al., 2010); peppermint essential oil did not change VH, CD, or VH/CD ratio (Emami et al., 2012). Kiczorowska et al. (2016) found that *Boswellia serrata* resin did not change VH or mucosal thickness but reduced CD and increased VH/CD ratio. Since most of the nutrient absorption takes place in the jejunum, the increase in VH, VW, and VA with FEO supplementation may have supported greater nutrient absorption, increasing growth. Furthermore, a thick layer of muscularis mucosa may promote greater nutrient absorption by increasing villi mobility, nutrient advancement, and upsetting digestion. Therefore, an increase in MMT is considered to have a positive effect. Regarding the effects of FEO on ileum histology, we found that FEO increased VH, VA, and MMT but had no effect on CD, VH/CD ratio, VW, VA, or TMT. Similarly, a thymol + carvacrol (1:1) mixture increased VH, VH/CD ratio, and muscular thickness but did not change VW, VA, CD, or mucosal thickness (Hashemipour et al., 2013). Previous research on ileum morphological measurements demonstrated that garlic meal increased VH, CD, and VH/CD ratio (Adibmoradi et al., 2006); dried fermented ginger did not affect VH or VA (Incharoen et al., 2010); a mixture of PFAs increased VH but did not change CD, VH/CD ratio, or mucus layer (Tsirtsikos et al., 2012); another PFA mixture did not change VH, CD, or VH/CD ratio (Fascina et al., 2017). Most of the microbial population is located in the ileocecal regions. Therefore, microbial composition may affect ileal morphology. In this context, it can be said that the scarcity of pathogenic microorganisms due to the antimicrobial activity of FEO may have improved ileal morphology.

### Small intestinal microflora

Considering the effect of FEO on jejunum microflora composition, it was found an insignificant difference between the groups in terms of *E. coli* O-157:H7, *S. aureus*, *Salmonella* spp., and *C. perfringens* counts. However, *Lactobacillus* spp. counts in the jejunum increased in parallel with higher FEO levels. Erhan et al. (2012) reported that 0.25% or 0.50% pennyroyal reduced *E. coli* count in the jejunum of broiler chickens in parallel with higher levels but significantly increased lactic acid bacteria count. Tzora et al. (2017) examined the effect of oregano essential oil, atapulgite, benzoic acid, and their blend on the microbial composition of the jejunum; the authors found that none of the applications affected total bacteria, *Lactobacillus* spp., or *C. perfringens* count, while the mixture group increased *Enterobacter* count (Tzora et al., 2017). Burt (2004) attributed the antimicrobial mode of action of many PFAs to the hydrophobicity of these phenolic compounds and their ability to penetrate the bacterial cell membrane, leading to cell membrane disruption, ion leakage, and eventually cell death. Di Pasqua et al. (2006) attributed the antimicrobial effect to changes in the fatty acid composition of bacterial cell membranes. Changes in fatty acid composition can affect the survival of microorganisms. Therefore, FEO may have altered the fatty acid composition of the pathogenic microorganisms, leading to an increase in beneficial bacteria. Thus, it can be concluded that FEO suppresses the growth of pathogens and stimulates the growth of beneficial bacteria. Moreover, Pirgozliev et al. (2019) reported that the carbonyl groups, anethole and fencone, in FEO bind to cellular membrane proteins and induce degradation of the lipid fraction of the plasma membrane of microorganisms, leading to changes in membrane permeability and leakage of intracellular substances.

Regarding the efficacy of FEO on the microbial population in the ileum, there was no difference between the groups in terms of *E. coli* O-157:H7, *Salmonella* spp., or *C. perfringens* counts. However, adding FEO significantly reduced *S. aureus* count. *Lactobacillus* spp. count increased in parallel with higher FEO levels. Based on previous studies, many PFAs caused differences in the ileum microorganism counts in broiler chickens. Safaei-Cherehh et al. (2018) reported that fennel extract reduced *E. coli* count but did not affect *Lactobacillus s*pp. count, which is contrary to our results. Çakmak et al. (2017) found that 0.75 and 1.5 g/kg sumac powder reduced *E. coli* count but increased lactic acid bacteria count in broiler chickens housed at different stocking densities. İpçak and Alçiçek (2019) reported that 150 mg/kg capsicum oleoresin, cinnamaldehyde, carvacrol, or their mixtures (1:1:1) did not change *S. aureus*, *C. perfringens*, or *Salmonella* spp. count but significantly increased *Lactobacillus* spp. count. According to other research on ileum microbial composition, 300 mg/kg oregano, garlic essential oil, and their mixtures reduced *Clostridium* spp. count (Kırkpınar, et. al., 2011); *Forsythia suspensa* extract, berberine, or their mixtures did not change *E. coli*, *Lactobacillu*s spp., or *Bifidobacter* counts (Zhang et al., 2013); thymol + carvacrol mixture (200 mg/kg) increased *Lactobacillus* spp. and *Bifidobacter* counts but did not reduce *E. coli* count (Hashemipour et al., 2013). These varying results may be attributed to the type of plant used, its parts, the phytochemical component it contains, the level of application, and the sensitivity of the microorganism species and strains. FEO exerts a bacteriostatic or bactericidal effect, possibly by causing the breakdown of the outer membranes of pathogenic microorganisms and leakage of cell contents. This may have increased the number of beneficial lactic acid-producing bacteria in the intestinal microflora. *Lactobacillus* spp. may also have promoted bird growth by lowering pH, increasing both essential oil activity and enzyme activity, and inhibiting pathogen growth. Overall, it can be said that FEO positively affected the intestinal microbiota ecosystem.

### Small intestine transcriptome profile

All biological processes in broiler chickens depend on the flow of genetic information (DNA-RNA-protein) in the central dogma. Although this information flow is controlled by the genetic structure, nutrients in the diet can regulate gene transcription. Therefore, the upregulation or downregulation of genes can alter the structure, activity, and amount of proteins that have various physiological functions, such as structural, mechanical, biochemical, cell signal transduction, and storage in the cell, which are of primary importance for life (Siddique et al., 2009; Sales et al., 2014). Ghasemian et al. (2020) reported that FEO is rich in anise aldehyde, which interferes with molecular targets in cells. Kim et al. (2010) investigated broiler chickens fed carvacrol (5 mg/kg), cinnamaldehyde (3 mg/kg), and capsicum oleoresin (2 mg/kg) and their high-throughput microarray analysis revealed that most of the genes with a 2-fold or higher change were associated with metabolic pathways, while most of the altered gene expression in the capsicum oleoresin group was associated with lipid metabolism, small molecule biochemistry, and cancer. Lillehoj et al. (2011) used the same experimental design and the same phytochemicals in a coccidiosis disease model by orally infecting one-day-old broiler chicks with *E. acervulina*, and found that most transcripts were observed in the capsicum oleoresin group, and most of these genes were related to metabolism and immunity. Research showed that cinnamaldehyde-induced genes were related to antigen presentation, humoral immune response, and inflammation. In another study on broiler chickens infected with *E. acervulina*, 1.810 transcripts (677 up, 1.133 down) were obtained in the intestinal intraepithelial lymphocytes of broiler chickens fed on anethole (15 mg/kg), the main bioactive component of FEO, compared to the positive and negative control groups, and the biological function of most of these transcribed genes was related to the inflammatory response (Kim et al., 2013a).

Sabino et al. (2018a) utilized the RNA-Seq technology and found that adding oregano aqueous extract to diets altered the expression of 129 genes in the liver of broiler chickens, most of which were downregulated genes involved in fatty acid metabolism and insulin signaling pathways. The same researchers conducted an RNA-Seq analysis of jejunum epithelial cells and reported that 280 genes were differentially expressed in broiler chickens fed olive mill wastewater; most of the upregulated genes were involved in the regulation of viral processes, while the downregulated genes were mostly related to the peroxisome proliferator activator receptor (fatty acid binding) signaling pathway, lipid metabolism, cholesterol, and steroid biosynthesis (Sabino et al., 2018b). Besides, the secondary metabolites, propyl thiosulfinate, and a compound containing propyl thiosulfinate oxide (Kim et al., 2013b) in intestinal intraepithelial lymphocytes and inulin in the liver tissue (Sevane et al., 2014), have been reported to alter the gene expression profile. Our analysis of global gene expression profiles in small intestinal tissues revealed that most of the upregulated genes were associated with catalytic activity, binding, transcription regulators, transcription factors, anatomical structure, cellular development, and protein binding activity regulators. These results showed that FEO could modulate the intestinal transcriptome profile of broiler chickens through different pathways to improve animal production efficiency. The different findings between studies could be attributed to the fact that due to the antimicrobial activity of FEO, less exposure of the organism to pathogenic bacteria or their toxic metabolites results in less need for immune defense activity in the gut; thus, protein production is allocated to different biological functions, such as growth or cellular development, as opposed to the inflammatory response with immune modulators (Steiner, 2006; Zeng et al., 2015a). Furthermore, it was found that most of the upregulated genes, especially in the low-dose (50 or 100 mg/kg) FEO-supplemented groups, belonged to the gene-specific transcriptional regulator or DNA-binding transcription factor protein class. Regulation of gene expression is governed by TFs (Duran et al. 2016). In other words, TFs determine how cells function and respond to nutrient changes in metabolically active organs such as the liver, intestine, and adipose tissue (Siddique et al., 2009). Thus, TFs may promote growth by binding to sequence-specific genes that are specifically responsible for growth, development, and gut health. According to our analyses, most of the upregulated genes in the high-level (200 or 400 mg/kg) FEO-supplemented groups belonged to the enzyme activity molecular function class. Therefore, the improved feed conversion ratio in the FEO-supplemented groups can be explained by the increased expression of genes responsible for catalytic/enzyme activity.

Based on our qRT-PCR analysis of IL10, MUC2, CLDN5, and CASP3 genes, only the change in IL10 was significant over the control group, with the highest change observed in the FEO100 group. IL-10 is an anti-inflammatory cytokine that inhibits the overexpression of nuclear factor kappa B (**NFκB**) and other pro-inflammatory cytokines (interferon-gamma [**IFN-γ**], IL-2, tumor necrosis factor-alpha [**TNF-α**]) that modulate immune responses and the exaggerated proliferation of various immune cells, such as neutrophils, macrophages, and natural killer cells, preventing the immune system from damaging vital cells and ensuring homeostasis. Furthermore, in the event of a pathogenic bacterial, parasitic, or viral attack, this class of cytokines can only suppress the immune system’s ability to fight these agents, allowing the organism to benefit more from the specific efficacy of antibiotics or antibiotic-like additives, such as FEO. Otherwise, a storm of cytokine release can occur, which can cause damage to healthy tissues and organs. On the other hand, in the absence of any stress factor, the FEO-mediated increase in IL-10 expression inhibits pro-inflammatory genes, thereby saving the cell’s energy. This may result in increased growth and development in the organism. Therefore, our findings regarding BWG may be associated with the utilization of this increased energy in growth. In parallel with our results, researchers reported that the addition of probiotics (*Bacillus subtilis*) (Lee et al., 2010) and anethole (Kim et al., 2013a) to broiler diets increased the expression of the IL-10. Korver (2012) reported that trans-anethole, which is responsible for antimicrobial activity, may reduce the activation of the inflammatory response and thus the associated growth-suppressive effects. Indeed, Yu et al. (2022) found that trans-anethole inhibited the expression of intestinal pro-inflammatory cytokines, including IL-1b, IL-8, IFN-γ, and TNF-α, but increased jejunal IL-10 and secretory immunoglobulin A concentrations in broiler chickens with subclinical NE. They also reported that trans-anethole ameliorated subclinical NE in broilers by causing a significant reduction in intestinal NF-kappa-B inhibitor alpha (**IkBα**) degradation and NF-κB translocation. The addition of a mixture of citrus peel, oregano, and aniseed (40 mg/kg) essential oils to the diet of piglets results in anti-inflammatory effects by reducing NF-κB and TNF-α gene expression (Kroismayr et al., 2008). In another study, oregano essential oil containing 81.92% carvacrol decreased the expression of mitogen-activated protein kinase and NF-κB immune response-modulating pathways and pro-inflammatory cytokines IL-1β, IL-6, IFN-α, TNF-α, and monocyte chemotactic protein-1 (MCP-1), demonstrating the anti-inflammatory effects of thyme essential oil in weaned pigs (Zou et al., 2016). Hence, adding FEO to broiler diets is predicted to reduce the need for immune defense activity in the intestine due to its antimicrobial effect, thereby allocating nutrients toward growth rather than immune defense.

In conclusion, based on its antimicrobial properties, adding FEO to diets improved broiler performance, promoted carcass yield and organ health, modulated small intestinal morphology and intestinal microflora, and modified the small intestinal transcriptome profile. The addition of FEO to feed as an alternative AGP in broiler diets is concluded to be effective, and the ideal level in terms of feed input cost-efficiency is 100 mg/kg FEO. Still, the application of nutrigenomic technologies in broiler nutrition is quite new. Therefore, researchers need to improve their technical knowledge to overcome both the complexity of the enormous datasets obtained and the experimental and computational difficulties, and more studies should be carried out for genome-based nutrition.

## Abbreviations

AGPs: antibiotics as growth promoters
AMR: antimicrobial resistance
BW: body weight
BWG: body weight gain
CD: crypt depth
EPEF: European Production Efficiency Factor
FCR: feed conversion ratio
FEO: fennel seed essential oil
FEO50: group supplemented with 50 mg FEO per kilogram of diet
FEO100: group supplemented with 100 mg FEO per kilogram of diet
FEO200: group supplemented with 200 mg FEO per kilogram of diet
FEO400: group supplemented with 400 mg FEO per kilogram of diet
FI: feed intake
IFN-γ: interferon-gamma
IkBα: NF-kappa-B inhibitor alpha
IL-10: interleukin 10
MBC: minimum bactericidal
MFC: minimum fungicidal
MIC: minimum inhibitory concentrations
MMT: muscularis mucosa thickness
mRNA: messenger RNA
NFκB: nuclear factor kappa B
PANTHER: Protein Analysis Through Evolutionary Relationships
PCA: principal component analysis
PFAs: phytogenic feed additives
qRT-PCR: quantitative real-time polymerase chain reaction
RMA: robust multichip analysis
SST-RMA: signal space transformation-robust multiarray analysis
TAC: Transcriptome Analysis Console
TF: transcription factor
TMT: tunica mucosa thickness
TNF-α: tumor necrosis factor-alpha
VA: villus area
VH: villus height
VW: villus width
